# HDL inflammatory index correlates with and predicts severity of organ failure in patients with sepsis and septic shock

**DOI:** 10.1371/journal.pone.0203813

**Published:** 2018-09-14

**Authors:** Faheem W. Guirgis, Sunita Dodani, Christiaan Leeuwenburgh, Lyle Moldawer, Jennifer Bowman, Colleen Kalynych, Victor Grijalva, Srinivasa T. Reddy, Alan E. Jones, Frederick A. Moore

**Affiliations:** 1 Department of Emergency Medicine, University of Florida College of Medicine-Jacksonville, Jacksonville, FL, United States of America; 2 Department of Medicine, Eastern Virginia Medical School, Norfolk, VA, United States of America; 3 Department of Aging and Geriatrics, University of Florida, College of Medicine, Gainesville, FL, United States of America; 4 Department of Surgery, University of Florida College of Medicine, Gainesville, FL, United States of America; 5 Department of Medicine, Molecular & Medical Pharmacology, UCLA School of Medicine, Los Angeles, CA, United States of America; 6 Department of Emergency Medicine, University of Mississippi College of Medicine, Jackson, MS, United States of America; University of Milano, ITALY

## Abstract

**Objective:**

High density lipoprotein (HDL) is important for defense against sepsis but becomes dysfunctional (Dys-HDL) during inflammation. We hypothesize that Dys-HDL correlates with organ dysfunction (sequential organ failure assessment (SOFA) score) early sepsis.

**Methods:**

A prospective cohort study of adult ED sepsis patients enrolled within 24 hours.

**Results:**

Eighty eight patients were analyzed. Dys-HDL (expressed as HDL inflammatory index (HII)) correlated with SOFA at enrollment (r = 0.23, p = 0.024) and at 48 hours (r = 0.24, p = 0.026) but HII change over the first 48 hours did not correlate with change in SOFA (r = 0.06, p = 0.56). Enrollment HII was significantly different in patients with most severe organ failure (2.31, IQR 1.33–5.2) compared to less severe organ failure (1.81, IQR 1.23–2.64, p = 0.043). Change in HII over 48 hours was significantly different for in-hospital non-survivors (-0.45, IQR-2.6, -0.14 p = 0.015) and for 28-day non-survivors (-1.12, IQR -1.52, 0.12, p = 0.044). In a multivariable linear regression equation (R^2^ = 0.13), for each unit HII increase, 48-hour SOFA increased by 0.72 (p = 0.009).

**Conclusion:**

HII correlated with SOFA and predicted 48-hour SOFA score in early sepsis. Future studies are needed to delineate potential mechanisms.

**Trial registration:**

NCT02370186. Registered February 24, 2015.

## Introduction

The pathobiology of sepsis is both complex and multifaceted. The prevailing theory of infection triggering a dysregulated immune response which leads to microcirculatory derangements and the clinical syndrome of organ failure and shock is likely to be correct, at least in part.[1] As the disease progresses, it is sustained by both host and pathogen elements which lead to cell signaling processes and genes which activate inflammatory pathways, adaptive immunity, and hypermetabolism. Recently, it has been recognized that both inflammatory and immunosuppressive responses occur concomitantly, with immunosuppression being prevalent in the later stages, which leads to secondary and opportunistic infections.[2–5]

Less well-studied, however, is the role that circulating lipids and lipoproteins may play in sepsis. We and others have reported that levels of both high density lipoprotein (HDL-C) and low density lipoprotein (LDL-C) cholesterol are dysregulated during sepsis.[6–8] HDL is postulated to protect against sepsis by its ability to: 1) clear bacterial toxins (lipopolysaccharide and lipoteichoic acid) via reverse cholesterol transport, 2) modulate innate cellular immunity and prevent release of inflammatory cytokines, and 3) transport cholesterol to the adrenal glands for steroid synthesis during septic stress.[9–13] LDL is also considered protective against sepsis for its ability to clear bacterial toxins, and as a provider of substrate for steroid synthesis.[9] However, during acute or chronic inflammation, lipoprotein particles become oxidized; and when HDL-C particles become oxidized, they can become dysfunctional and pro-inflammatory, resulting in dysfunctional HDL (Dys-HDL). This process results in HDL which is not only incapable of performing its protective functions, but may also continue to propagate inflammation and tissue damage during sepsis.

For these reasons, we sought to characterize the role that Dys-HDL may play in the pathobiology of sepsis-associated organ dysfunction. To do this, our primary objective was to measure Dys-HDL in patients with early sepsis and septic shock presenting to the emergency department, and to determine if Dys-HDL levels correlate with severity of organ failure as measured by the sequential organ failure assessment (SOFA) score. Secondary objectives were to determine the association between early Dys-HDL levels and in-hospital and 28-day mortality. We hypothesized that early Dys-HDL levels would correlate with SOFA score, and that change in Dys-HDL levels would correlate with change in SOFA scores in the first 48 hours.

## Methods

### Study setting, design and patient selection

Critically ill adult patients (age ≥ 18 years) presenting to the University of Florida (UF) Health Jacksonville emergency department (ED) with sepsis or septic shock were approached for enrollment in this prospective, observational cohort study. Patients meeting criteria were enrolled within 24 hours of ED presentation if they were being treated with an evidence-based guideline treatment bundle for sepsis. The UF Health Jacksonville ED is a high acuity, academic, urban ED which treats approximately 95,000 patients per year. The research protocol was approved by the UF College of Medicine, Jacksonville Institutional Review Board (IRB# 201702454) and all experiments were performed in accordance with relevant guidelines and regulations.

Study inclusion criteria were: a) infection with > 1 SIRS criteria, b) lactate ≥ 2 mmol/L, c) SOFA score ≥ 4, and d) sepsis as the primary diagnosis for admission. Exclusion criteria were: 1) pregnancy, 2) lack of valid consent, 3) familial or genetic disorders of lipid metabolism, 4) active seizure, and 5) cardiopulmonary resuscitation prior to enrollment. Patients were consented as per IRB requirements.

### Measurements and interventions

Study data were prospectively collected and included age, sex, race, place of residence, suspected source of infection, and comorbidities such as diabetes mellitus, chronic obstructive pulmonary disease (COPD), end-stage renal disease (ESRD), Human Immunodeficiency Virus (HIV) status, active cancer, and organ transplant. Physiologic and treatment variables collected were lactate levels, SOFA score, triage and enrollment vital signs, timing of antibiotics, volume of intravenous fluids administered in the first six and 24 hours, vasopressor use and duration, mechanical ventilation use, central venous pressures (CVP), urine output in the first six hours. Additional data included familial disorders of lipid metabolism, statin use, admission disposition, hospital length of stay (LOS), and ICU LOS. At 48 hours, repeat clinical assessments were performed including repeat vital signs, hemodynamic and ventilator requirements, and SOFA score. Glasgow Coma Scale (GCS) scores were assessed prospectively at both time points by trained study physicians. Chart reviews after enrollment were performed to confirm source of infection and sepsis diagnosis, culture results, ICU and hospital LOS, and outcome and discharge disposition.

Blood sampling occurred at baseline enrollment (within 24 hours of ED arrival) and 48 hours after enrollment and included total cholesterol levels, HDL-C, LDL-C, triglycerides, laboratory tests for SOFA score calculation, and Dys-HDL testing. At both time points serum and plasma samples were obtained, processed, and frozen at -80°C until further testing. The study protocol required blood to be drawn within four hours of planned collection times.

Dys-HDL was quantitated using a cell-free assay and expressed as HDL Inflammatory Index (HII) as in previous studies.^8^ Briefly, the cell free assay for Dys-HDL requires HDL isolation from blood samples using dextran sulfate precipitation and LDL prepared from a normal donor. Oxidation of LDL resulted in the release of dichlorofluorescein (DCFH) which was quantitated with an excitation wavelength of 485 nm, emission wavelength of 530 nm, and cutoff of 515 nm. After incubating experimental (patient HDL plus control LDL) and control samples (control LDL only) with dichlorofluorescein, the ability of sample HDL to protect LDL from oxidation was quantitated by the decline in fluorescence and expressed as a ratio of the fluorescence released, the HII. The HII, used to quantitate Dys-HDL, was calculated by normalizing the cell-free assay values obtained for LDL alone as 1.0. If addition of a test HDL along with LDL resulted in an HII of 1.0 or greater, the test HDL was classified as pro-inflammatory (Dys-HDL). Conversely, if addition of a test HDL to LDL resulted in a HII of less than 1.0, the test HDL was classified as anti-inflammatory. Values for intra-assay and inter-assay variability are 5.3±1.7% and 7.1±3.2%, respectively.[14]

### Outcomes, data analysis, and sample size justification

The primary outcome of the study was severity of organ failure expressed by the SOFA score at enrollment, 48 hours after enrollment, and the change in SOFA scores over that time. As HII data do not follow a normal distribution, Spearman’s correlations were used to determine the correlation between HII and SOFA score at each time. Univariate analyses of multiple covariates were performed using Student's T test for numerical, normally distributed data and Wilcoxon rank-sum was used for numerical, non-normally distributed data. A multivariable linear regression model was also created to assess the predictive ability of enrollment HII for 48-hour SOFA score.

To accept the alternative hypothesis that change in Dys-HDL correlates with change in SOFA score, and based on our estimated effect size, we calculated that a sample size of 85 patients with two measurements of Dys-HDL and SOFA were needed to detect a correlation coefficient r of 0.30 with a power of 0.80 at a 0.05 significance level. Therefore, based on the estimated early mortality rate of 17% at our institution, we planned to enroll 105 patients to account for potential deaths which could occur in the first 48 hours to achieve adequate power.

## Results

**Fig 1:**
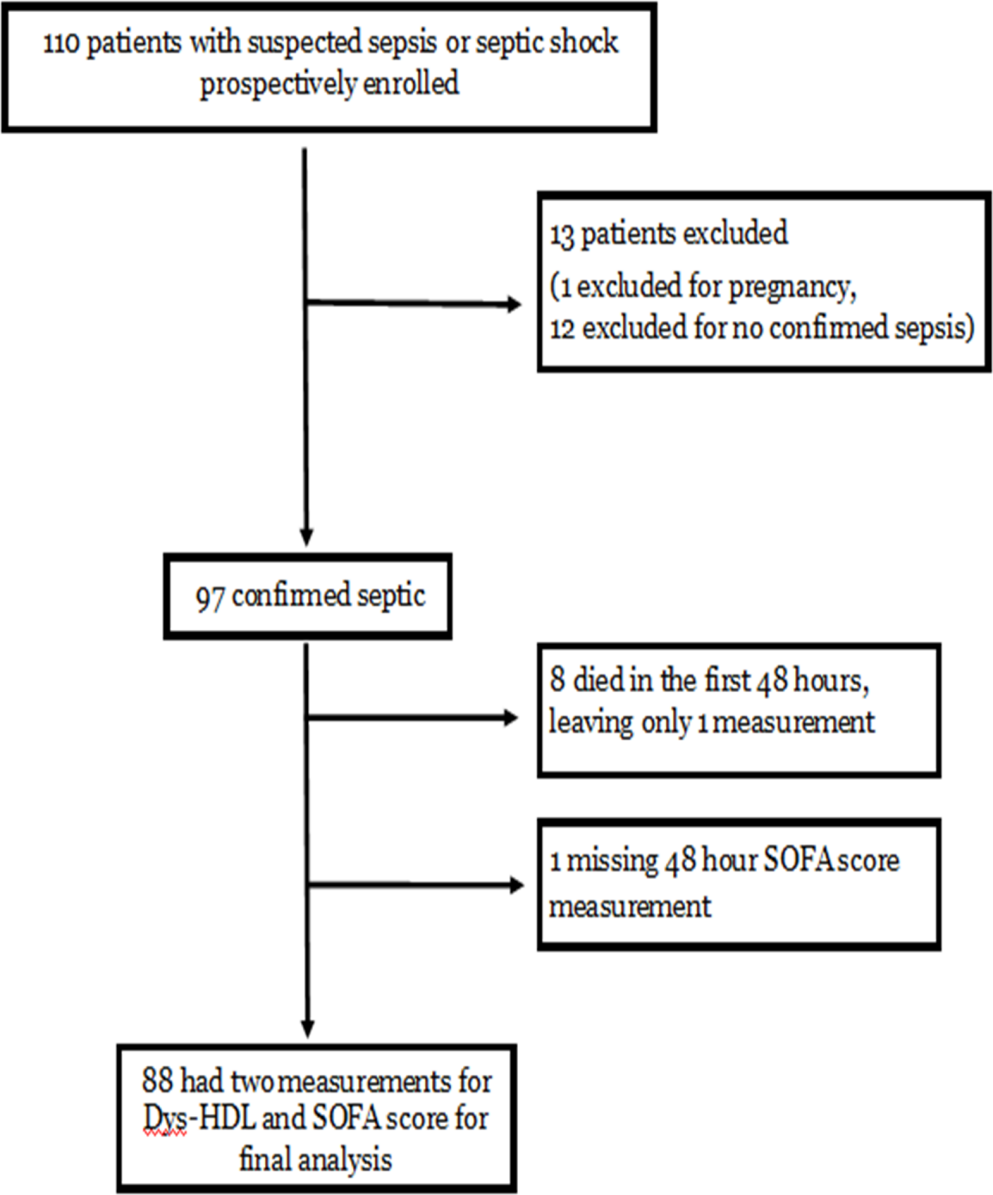
Patient enrollment flow diagram.

There were 110 patients prospectively enrolled in the study **(Fig 1).** Thirteen patients were later excluded, leaving 97 patients who were confirmed septic and had enrollment measures of Dys-HDL and SOFA score. Of these, eight patients died within the first 48 hours, and one patient did not have laboratory measurements for repeat SOFA score, leaving 88 patients with two measures of Dys-HDL and SOFA score.

For all enrolled study patients with confirmed sepsis (N = 97), patients were generally older with a mean age of 64 years, and there were slightly more males (54%) than females (46%). Race was almost evenly distributed between White and Black, and there were no Asian or Hispanic patients. The top three sources of infection were pulmonary (42%), urinary tract (34%), and intra-abdominal (12%) and the most common comorbidity was diabetes mellitus (42%). Most study patients were critically ill with 33% requiring mechanical ventilation on enrollment, 80% directly admitted to an ICU, and a median ICU LOS of 4 days (IQR 2–7). In-hospital mortality was 20% and 28-day mortality was 31%. Of the 80% (78/97) of patients who survived hospital admission, 42% were discharged to home or to a rehabilitative facility, and 38% were discharged to a nursing home, hospice, or a long-term acute care facility. Only 69% (67/97) of patients survived to 28 days. Complete descriptive characteristics of the study population are presented in **Table 1.**

**Table 1:** Study patient demographics, source of sepsis, comorbidities, early clinical features, early sepsis management and outcomes and disposition. SD = standard deviation. IQR = interquartile range. NH = nursing home. ICU = intensive care unit. LOS = length of stay.

Variable, N = 97	Value
**Demographics**	
Age, mean (SD)	64 years (14)
Male, % (N)	54% (52/97)
White, % (N)	52% (50/97)
Black, % (N)	48% (47/97)
**Source of sepsis**, % (N)	
Pulmonary	42% (41)
Urinary	34% (33)
Intra-abdominal Skin/Soft-tissue	12% (12) 6% (6)
Blood/Indwelling catheter Unknown Source	5% (5) 1% (1)
**Comorbidities**, % (N)	
Diabetes mellitus	44% (43)
Chronic obstructive pulmonary disease	24% (23)
End stage renal disease	11% (11)
Active cancer Organ transplant	10% (10) 2% (2)
Human Immunodeficiency Virus positive	3% (3)
Admitted from nursing home	20% (19)
**Early Clinical Features**	
Initial systolic blood pressure, mm Hg, mean (SD)	115 (34)
Initial mean arterial pressure, mm Hg, mean (SD)	78 (16)
Initial heart rate, beats/min, mean (SD)	107 (26)
Initial temperature, °F, mean (SD)	99.5 (2.3)
Initial oxygen saturation, mm Hg, mean (SD) Initial respiratory rate, breaths/min, mean (SD)	94 (11) 24 (10)
Initial Lactate, mmol/L, mean (SD) Repeat Lactate, mmol/L, mean (SD)	3.7 (1.6) 3.4 (1.8)
SOFA score, median (IQR)	7 (5–10)
**Early Sepsis Management**	
Intravenous fluids in the first 6 hours, mL, median (IQR)	3000 (2000–4750)
Intravenous fluids in the first 24 hours, mL, median (IQR)	5000 (3000–7000)
Time to antibiotic administration, minutes, mean (SD)	146 (112)
Mechanical ventilation use on enrollment, % (N)	33% (32)
Vasopressor use on enrollment, % (N)	42% (41)
Duration of vasopressor use, hours, median (IQR)	39 (22–67)
**Outcomes and Disposition**	
Initial ICU admission, % (N)	80% (78)
ICU LOS, days, median (IQR)	4 (2–7)
Hospital LOS, days, median (IQR)	8 (5–13)
In-hospital mortality, % (N)	20% (19)
Disposition at discharge–Home or Rehab facility	42% (41)
Disposition at discharge–NH, hospice, LTAC	38% (37)
28-day mortality, % (N)	31% (30)

For patients with two assessments who could be assessed for the primary outcome (N = 88), Dys-HDL (HII) significantly correlated with SOFA scores at enrollment (r = 0.23, p = 0.024) and at 48 hours (r = 0.24, p = 0.026) but change in HII over the first 48 hours did not correlate with change in SOFA (r = 0.06, p = 0.56) (**Table 2)**. Enrollment HII was also significantly different for patients with the highest quartile SOFA score upon enrollment (2.31, IQR 1.33–5.2) in comparison to those in the bottom three quartiles (1.81, IQR 1.23–2.64, p = 0.043). HII at 48 hours and change in HII over 48 hours were not significantly different by severity of organ failure **(Table 3).**

**Table 2:** Spearman’s correlations for dysfunctional HDL (Dys-HDL), expressed as HDL Inflammatory Index (HII), and SOFA score at enrollment and 48 hours (48h) after enrollment. HDL, high density lipoprotein.

Dys-HDL Variable	Dys-HDL (median, IQR)	SOFA Score (median, IQR)	SOFA Score (Spearman’s rho)	P-value
Dys-HDL (HII) at enrollment, N = 97	1.9 (1.32, 2.68)	7 (5,10)	0.23	**0.0237**
Dys-HDL (HII) at 48h, N = 88	1.67 (1.06, 2.4)	5 (1,7.5)	0.24	**0.0258**
			**Delta SOFA Score** (48h –enrollment)	
Delta Dys-HDL (48h –enrollment), N = 88	-0.14 (-0.51, 0.24)	-2 (-4, 0)	0.06	0.56

**Table 3:** Lipid levels and dysfunctional HDL (Dys-HDL) levels at enrollment and 48 hours after enrollment by top quartile SOFA score and SOFA increase over 48 hours. Student’s T test was performed for HDL-C, LDL-C, and lactate; Wilcoxon’s rank sum test was used for Dys-HDL and ICU stay; and Pearson’s Chi Square test was used for mechanical ventilation and vasopressors use comparisons. HDL, high density lipoprotein. SOFA, sequential organ failure assessment. LDL, low density lipoprotein. HII, HDL inflammatory index. SD, standard deviation. IQR, interquartile range.

**Variable**	**Enrollment SOFA**		**P value**
**Enrollment value**	**Bottom 75% (N = 74)**	**Top 25% (N = 23)**	
Dys-HDL level (HII), median (IQR)	1.81 (1.23–2.64)	2.31 (1.33–5.2)	**0.043**
HDL-C level (mg/dL), mean (SD)	28.5 (19.1)	27.0 (20.0)	0.74
LDL-C level (mg/dL), mean (SD)	51.6 (26.6)	42.8 (24.9)	0.17
Total Cholesterol (mg/dL), mean (SD)	106.0 (33.9)	95.3 (28.9)	0.19
Lactate (mmol/L), mean (SD)	3.26 (SD 1.68)	4.03 (SD 2.7)	0.14
Mechanical Ventilation, % (n/total)	34% (25/74)	87% (20/23)	**<0.001**
Vasopressor Use, % (n/total)	30% (22/74)	83% (19/23)	**<0.001**
ICU stay, median days (IQR)	4 (2–6)	5 (3–8)	0.19
**48-hour value**	**48-hour SOFA**		**P value**
	**Bottom 75% (N = 66)**	**Top 25% (N = 31)**	
Dys-HDL level (HII), median (IQR)	1.63 (1.05–2.4)	1.74 (1.1–2.4)	0.73
HDL-C level (mg/dL), mean (SD)	22.2 (17.5)	12.5 (9.4)	**0.015**
LDL-C level (mg/dL), mean (SD)	57.3 (23.6)	39.3 (20.5)	**0.003**
Total Cholesterol (mg/dL), mean (SD)	109.8 (30.9)	81.9 (23.8)	**<0.001**
Vasopressor Use at 48 hours, % (n/total)	5% (3/66)	59% (13/22)	**<0.001**
**Delta (48 hour–enrollment)**	**Delta SOFA**		**P value**
	**Decrease (N = 76)**	**Increase (N = 21)**	
Dys-HDL (HII), median (IQR)	-0.14(-0.51, 0.23)	-0.19 (-0.57, 0.4)	0.81
HDL-C (mg/dL), mean (SD)	-8.3 (11.9)	-10.8 (15.4)	0.53
LDL-C (mg/dL), mean (SD)	3.7 (21.9)	-10.8 (21.6)	**0.044**
Total Cholesterol (mg/dL), mean (SD)	0.41 (25.6)	-17.1 (21.4)	**0.035**

For the secondary outcomes, HII upon enrollment was not significantly different for in-hospital non-survivors (2.19, IQR 1.32–3.6) compared with survivors (1.86, IQR 1.23–2.65, p = 0.22). However, enrollment HII was significantly different in 28-day non-survivors (2.47, IQR 1.49–3.6) compared with survivors (1.72, IQR 1.19–2.47, p = 0.016). Interestingly, the delta HII (48 hour HII–enrollment HII) was significantly different for in-hospital non-survivors (-0.45, IQR-2.6, -0.14) compared with survivors (0.01, IQR -0.46, 0.26, p = 0.015), as well as for 28-day non-survivors (-1.12, IQR -1.52, 0.12) compared with survivors (.01, -0.45, 0.25, p = 0.044). This difference in delta HII indicates a greater drop in HII from enrollment to 48 hours (larger negative number) for delta HII in non-surviving patients.

Cholesterol levels also varied by outcome (**Tables 3 and 4**). Though enrollment HDL-C and LDL-C levels were not significantly different in patients with the highest severity of organ failure compared with the rest of the cohort, 48-hour HDL-C levels were significantly lower in patients with the highest quartile SOFA score at 48 hours (12.5 mg/dL, SD 9.4) compared to patients in the bottom three quartiles (22.2 mg/dL, SD 17.5, p = 0.015). The same was seen for 48-hour LDL-C levels which were significantly lower in patients with severe organ failure (39.3 mg/dL, SD 20.5) compared to the rest of the cohort (57.3 mg/dL, SD 23.6, p = 0.003). LDL-C levels were also significantly different at both enrollment (p = 0.018) and 48 hours (p = 0.002) in patients experiencing in-hospital death, but not for 28-day non-survivors. HDL-C levels were not significantly different at enrollment or 48 hours in patients experiencing in-hospital death or 28-day mortality.

**Table 4:** Dysfunctional HDL (Dys-HDL) and lipid levels at enrollment and 48 hours after enrollment by in-hospital death and 28-day mortality. Student’s T test was performed for HDL-C, LDL-C, and lactate; Wilcoxon’s rank sum test was used for Dys-HDL and SOFA score; and Pearson’s Chi Square test was used for mechanical ventilation and vasopressors use comparisons.HDL, high density lipoprotein. LDL, low density lipoprotein. HII, HDL inflammatory index. SD, standard deviation. IQR, interquartile range.

Variable	In-hospital Death		P value	28 Day Mortality		P value	
	Dead N = 19	Living N = 78		Dead N = 30	Living N = 67		
**Enrollment value**							
Dys-HDL (HII), median (IQR)	2.19 (1.32–3.6)	1.86 (1.23–2.65)	0.22	2.47 (1.49–3.6)	1.72 (1.19–2.47)		**0.016**
HDL-C (mg/dL), mean	25.2	28.8	0.47	23.7	29.7		0.15
LDL-C (mg/dL), mean	36.0	52.6	**0.018**	45.5	51.7		0.30
Total Cholesterol (mg/dL), mean	93.2	105.8	0.16	98.8	105.8		0.35
Lactate (mmol/L), mean	4.78	3.07	**0.001**	4.68	2.84		**<0.001**
Mechanical Ventilation, % (n/total)	14/19	31/78	**0.008**	26/66	19/30		**0.029**
Vasopressor Use, % (n/total)	12/19	29/78	**0.04**	23/66	17/30		**0.044**
SOFA, median (IQR)	11 (7–14)	6 (4–9)	**<0.001**	10.5 (5–12)	6 (4–9)		**0.001**
**48-hour value**							
Dys-HDL (HII), median (IQR)	1.66 (1.05–2.25)	1.68 (1.08–2.4)	0.70	2.05 (1.13–2.49)	1.59 (1.05–2.39)		0.34
HDL-C (mg/dL), mean	15.1	20.5	0.31	13.6	21.4		0.054
LDL-C (mg/dL), mean	32.5	55.8	**0.002**	43.8	56.3		**0.038**
Total Cholesterol (mg/dL), mean	81.8	106.1	**0.016**	87.4	108.4		**0.008**
Vasopressor Use, % (n/total)	6/11	10/77	**0.001**	9/21	7/66		**0.001**
SOFA, median (IQR)	9 (7–13)	5 (1–6)	**<0.001**	7 (6–10)	4 (1–6)		**<0.001**
**Delta value (48 hour–enrollment)**							
Delta Dys-HDL, median (IQR)	-0.45 (-2.6, -0.14)	0.01 (-0.46,0.26)	**0.015**	-1.12 (-1.52,0.12)	0.01 (-0.45,0.25)		**0.044**
Delta HDL-C (mg/dL), mean	-13.5	-8.0	0.16	-9.6	-8.3		0.68
Delta LDL-C (mg/dL), mean	-7.1	3.1	0.16	-3.2	3.5		0.24
Delta Total Cholesterol (mg/dL), mean	-17.0	0.40	**0.036**	-11.8	1.5		**0.042**

In a multivariable linear regression model calculated to predicted 48-hour SOFA score, a significant regression equation was found (F(2, 85) = 6.17, p 0.003), with an R^2^ of 0.13 **(Table 5)**. Enrollment active cancer (p = 0.027) and enrollment HII (p = 0.008) were significantly predictive. The predicted 48-hour SOFA score was equal to 3.18 + 0.72 (HII) so that SOFA increased by 0.72 points for each unit increase in HII. Similarly, 48-hour SOFA increased by 3.18 + 3.16 for patients having active cancer.

**Table 5:** Multivariable linear regression model for the prediction of 48-hour sequential organ failure assessment (SOFA) score. Predictor variables included in the model included diabetes mellitus, chronic obstructive pulmonary disease, active cancer, organ transplant recipient, HIV status, enrollment values for HDL inflammatory index (HII), LDL-C, HDL-C, serum lactate levels, and enrollment mechanical ventilation status.

Predictor	Coefficient	95% CI	P-value
Active Cancer	3.16	0.36–5.96	0.027
Enrollment HII	0.72	0.19–1.25	0.008

## Discussion

In this prospective observational cohort study, we have demonstrated a significant correlation and predictive ability for early Dys-HDL levels with severity of organ failure in adult patients with sepsis and septic shock. We also demonstrated a significant association between Dys-HDL levels and in-hospital death and between early cholesterol levels and severity of organ failure and death from sepsis. Overall, these data support the hypothesis that dysregulated lipoprotein metabolism may contribute to the pathogenesis of organ failure in sepsis.

Pre-clinical data have long demonstrated a role for lipid mediators in defense against sepsis. The ability for HDL to mediate the clearance of bacterial toxins during sepsis has been shown, where HDL has the ability to bind and move endotoxin from gram negative bacteria and lipoteichoic acid from gram positive bacteria via its main apolipoprotein, Apo-AI.[9–11,15,16] Similarly, LDL plays a role in bacterial toxin clearance.^9^ Recent attention has come to the potentially critical role of the LDL receptor on hepatocytes in this process where LDL-R knockout mice, which have reduced ability to clear LDL-C from the blood, also demonstrate reduced endotoxin clearance.[17] A critical link with the proprotein convertase subtilisin/kexin type 9 (PCSK9) molecule has also recently been established. PCSK9 binds and degrades the LDL receptor which results in increased LDL-C levels in the blood, and it has been shown that increased PCSK9 levels in human sepsis patients are associated with reduced endotoxin clearance in cultured human hepatocytes.[18,19] Our group has also shown that reduced LDL-C levels are a risk factor for the future development of sepsis.[20]

The presence of Dys-HDL in this study, as well as the associations between early Dys-HDL elevation with severe organ failure and death, may have biological implications. Dys-HDL elevation implies that HDL has become proinflammatory and lacks the ability to protect LDL from further oxidation.[14] Furthermore, Dys-HDL may potentiate the inflammatory response in sepsis, causing cellular damage and potentially influencing organ dysfunction. Though this was not directly demonstrated, another possible explanation for our findings is that oxidized lipids are being used by the body to combat bacteria, and therefore are being generated strategically as a defense mechanism. Dys-HDL elevation in this cohort may also be reflective of general inflammatory and oxidative stress during sepsis and be a measure of sepsis disease severity. Because the method of HDL isolation using dextran sulfate precipitation for the cell-free assay results in testing of HDL supernatant, it is possible that the elevated HII in our cohort was due to other oxidized molecules. However, the robustness of the cell-free assay has been demonstrated by our group previously.[14] Specifically, HDL isolated by agarose gel electrophoresis, fast performance liquid chromotography, and dextran sulfate precipitation all resulted in nearly identical fluorescence intensity when exposed to an oxidizing agent. We have also previously shown that HDL isolated by dextran sulfate precipitation yields almost no fluorescent signal by itself, indicating that the oxidized molecules in the supernatant do not appear to contribute significantly, though this was not specifically tested in the septic patients in this cohort.[14] Finally, the ability of test HDL from the cell-free assay to inactivate and prevent the formation of oxidized lipids has been previously shown to yield identical results to our established cell-based assay.[21]

Interestingly, though our findings show the predictive ability of Dys-HDL at enrollment for 48-hour organ failure, and demonstrate disproportionate enrollment Dys-HDL elevation in patients with more severe organ failure (Table 3) compared with less severe organ failure, Dys-HDL at 48 hours (HII) and delta HII did not show significant associations with organ failure. However, there were significant associations between delta HII and both in-hospital death and 28-day mortality. Enrollment Dys-HDL was also significantly different in 28-day non-survivors but not in in-hospital non-survivors. One potential explanation for these varied results and the fact that 48-hour Dys-HDL was not significantly associated with organ failure, is that lipids may have been eliminated from the body via the liver, or degraded due to upregulation of inflammatory cytokines and degradative enzymes (hepatic lipase, endothelial lipase, serum phospolipase A2). Future work will attempt to characterize these changes and define specific lipid and lipoprotein moieties on HDLs surface that may explain these variations over time.

Another unanswered question is the cause for the rapid decline in cholesterol levels that occurs in early sepsis, which is more exaggerated with increasing sepsis severity. Our findings that show an association between the severity of the drop in cholesterol levels with organ failure and death are similar to what others have described.[6,7,22] Though many possible explanations have been proposed, it is likely that no single explanation is sufficient. As previously mentioned, some have proposed that HDL-C and LDL-C may take part in the process of reverse cholesterol transport to mediate toxin clearance, and that HDL-C is transporting cholesterol to the adrenal glands for steroid production, resulting in their removal from circulation.[23,24] This is a scavenger receptor BI (SR-BI)-mediated process, and mice overexpressing SR-BI demonstrate the virtual disappearance of HDL from circulation.[23] This may be seen as contrary to our previous findings that HDL cholesterol efflux capacity is impaired in septic patients compared to healthy controls, however cholesterol efflux capacity is only the first limiting step in reverse cholesterol transport. It does not take into account hepatic SR-BI receptor activity or expression or cholesterol ester transfer protein-mediated hepatic cholesterol transfer that are necessary for the entire process.[25] Zimetti and colleagues recently studied 59 patients with an acute phase response, of whom 37 had infections, and noted that APR patients had changes in HDL composition (specifically the displacement of ApoA-I by serum amyloid A) and reduction in cholesterol efflux (specifically due to decreased activity of SR-BI and ABCG1), going against this hypothesis.[26] Our study is different from Zimetti’s however, in that our population of severe sepsis and septic shock patients was critically ill as nearly all patients had multiple organ failure (SOFA score median 7, IQR 5–10), and the overall in-hospital and 28-day mortality were 20% and 31%, respectively. Critically ill shock patients have also been shown to be in a state of lypolysis, as demonstrated in a study of 182 mechanically ventilated ICU patients where tissue microdialysis catheters were placed in femoral adipose tissue and demonstrated increased glycerol and free fatty acids.[27] In that study which, included a population of septic shock patients, increased triglycerides and reductions in total cholesterol, LDL-C, and HDL-C levels were noted. Finally, other proposed mechanisms explaining the acute reductions in HDL-C concentrations have been proposed including reduced HDL generation due to suppressed expression of the hepatic ABCA1 transporter,[28] increased phospholipase A_2_ activity resulting in lipolysis, [29] and increased serum amyloid A resulting in increased HDL clearance.[24,30]

This study has several potential clinical implications. The first depends on whether Dys-HDL elevation is indicative of sepsis pathobiology leading to organ dysfunction. If the mechanisms by which Dys-HDL elevation occurs can be elucidated, we may learn more about the genesis and clearance of oxidized lipids by the body. If there are protective processes that become dysfunctional or downregulated during sepsis, the potential to augment these pathways may hold novel sepsis therapeutic options such as increasing LDL clearance with PCSK9 inhibitors, administering lipid emulsions for toxin binding, and facilitating endogenous corticosteroid production. Future therapeutic options may also be poised to investigate mechanisms by which HDL function can be enhanced, as with the apolipoprotein mimetics which have been shown to hold some potential.[31–33]

This study had several limitations. This was a relatively small study, and while it was powered to detect a correlation with SOFA score, it was not powered to detect associations with mortality. Therefore, there may be associations with Dys-HDL and lipid levels with mortality and other outcomes, which were not observed due to lack of power. Second, while the associations of Dys-HDL with organ failure and death are very important, our study was not poised to explain the mechanisms for these associations. The cell free assay does not measure specific changes in oxidized lipids on the HDL particle, and as such cannot characterize which particular lipids and lipoproteins are altered. Future endeavors will attempt to characterize specific lipid and lipoprotein alterations on HDL. Finally, because HII may be sensitive to HDL concentration and to the presence of antioxidants, it cannot be excluded that changes in plasma HDL-C or in the content of pro or antioxidant components during sepsis could contribute to the observed difference in HII.

## Conclusion

In this study, we have demonstrated that early Dys-HDL levels correlate with and predict severity of organ failure in sepsis. Future work should focus on understanding the mechanisms by which Dys-HDL may contribute to organ failure during sepsis and the potential role for new treatment modalities.
